# The ecological diversification and evolution of Teleosauroidea (Crocodylomorpha, Thalattosuchia), with insights into their mandibular biomechanics

**DOI:** 10.1002/ece3.9484

**Published:** 2022-11-18

**Authors:** Michela M. Johnson, Davide Foffa, Mark T. Young, Stephen L. Brusatte

**Affiliations:** ^1^ Staatliches Museum für Naturkunde, Museum am Löwentor Stuttgart Germany; ^2^ Department of Geosciences Virginia Tech Blacksburg Virginia USA; ^3^ School of Geography, Earth and Environmental Sciences University of Birmingham Birmingham UK; ^4^ National Museum of Scotland Edinburgh UK; ^5^ School of GeoSciences, Grant Institute University of Edinburgh Edinburgh UK; ^6^ LWL‐Museum für Naturkunde Münster Germany

**Keywords:** Crocodylomorpha, ecology, functional morphology, mandibular biomechanics, Teleosauroidea, Thalattosuchia

## Abstract

Throughout the Jurassic, a plethora of marine reptiles dominated ocean waters, including ichthyosaurs, plesiosaurs and thalattosuchian crocodylomorphs. These Jurassic ecosystems were characterized by high niche partitioning and spatial variation in dietary ecology. However, while the ecological diversity of many marine reptile lineages is well known, the overall ecological diversification of Teleosauroidea (one of the two major groups within thalattosuchian crocodylomorphs) has never been explored. Teleosauroids were previously deemed to have a morphologically conservative body plan; however, they were in actuality morphofunctionally more diverse than previously thought. Here we investigate the ecology and feeding specializations of teleosauroids, using morphological and functional cranio‐dental characteristics. We assembled the most comprehensive dataset to date of teleosauroid taxa (approximately 20 species) and ran a series of principal component analyses (PC) to categorize them into various feeding ecomorphotypes based on 17 dental characteristics (38 specimens) and 16 functionally significant mandibular characters (18 specimens). The results were examined in conjunction with a comprehensive thalattosuchian phylogeny (153 taxa and 502 characters) to evaluate macroevolutionary patterns and significant ecological shifts. Machimosaurids display a well‐developed ecological shift from: (1) slender, pointed tooth apices and an elongate gracile mandible; to (2) more robust, pointed teeth with a slightly deeper mandible; and finally, (3) rounded teeth and a deep‐set, shortened mandible with enlarged musculature. Overall, there is limited mandibular functional variability in teleosaurids and machimosaurids, despite differing cranial morphologies and habitat preferences in certain taxa. This suggests a narrow feeding ecological divide between teleosaurids and machimosaurids. Resource partitioning was primarily related to snout and skull length as well as habitat; only twice did teleosauroids manage to make a major evolutionary leap to feed distinctly differently, with only the derived machimosaurines successfully radiating into new feeding ecologies.

## Graphical abstract

An investigation into teleosauroid functional morphology and ecological diversification. Using dental and mandibular characteristics, results suggest that teleosauroids displayed limited mandibular functional variability, aside from certain subgroups which exploited larger/harder prey items.

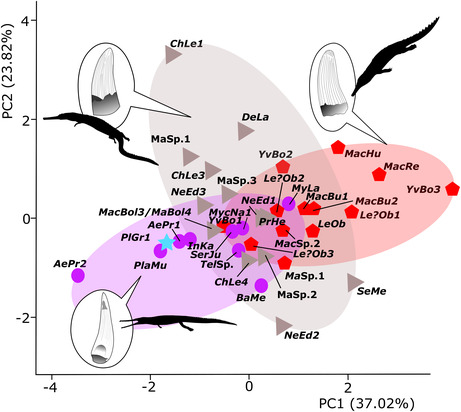

## 1 INTRODUCTION

Throughout the Mesozoic Era, a plethora of anatomically diverse marine reptiles dominated the oceans (Pyenson et al., 2014). During the Jurassic, three distantly related groups coexisted, sharing the top tiers of the marine trophic webs, ichthyosaurs, plesiosaurs (plesiosauroids and pliosaurids) and thalattosuchians (a group of extinct marine crocodylomorphs) (Benson & Druckenmiller, 2012; Foffa et al., 2018; Massare, 1987, 1988). Pioneering work by Massare (1987) assigned these extinct marine reptiles to broad ecological guilds (pierce, general, cut, smash, crunch, and crush) based on tooth morphology, but these were qualitative in nature and not universally accepted (Buchy, 2010). More recently, Foffa et al. (2018) examined the dentition of fossil marine reptiles over an approximately 18‐million‐year history of the Jurassic Sub‐Boreal Seaway (United Kingdom) to evaluate feeding ecology using a quantitative approach, validating the guild structure used by Massare (1987). Foffa et al. (2018)'s results showed that extinct marine reptile groups did not significantly overlap in guild space, indicating that dietary niche partitioning allowed many species to coexist.

While the dataset of Foffa et al. (2018) included a wide variety of marine reptile species, there were only a few representatives from Teleosauroidea. Teleosauroids are one of the two main groups within Thalattosuchia, a major radiation of marine crocodylomorphs that were abundant during the Jurassic and Early Cretaceous (the other being the metriorhynchoids, which by the Middle Jurassic gave rise to Metriorhynchidae, the first archosaurs to adopt a fully pelagic lifestyle) (Foffa & Young, 2014; Wilberg et al., 2019; Young et al., 2010). Teleosauroids were a near‐globally distributed and ecologically diverse clade that inhabited freshwater, brackish, lagoonal and deep‐water marine ecosystems (Buffetaut, 1982; Foffa et al., 2019; Johnson et al., 2017, 2019, 2020; Martin et al., 2016; Young et al., 2014). They used to be regarded as merely marine analogues of extant gavials, based on most species having dorsally directed orbits, an elongate and tubular snout and high tooth count, suggesting that they fed primarily on small, swift‐moving prey (Andrews, 1909, 1913; Buffetaut, 1982; Hua, 1999).

**FIGURE 1:**
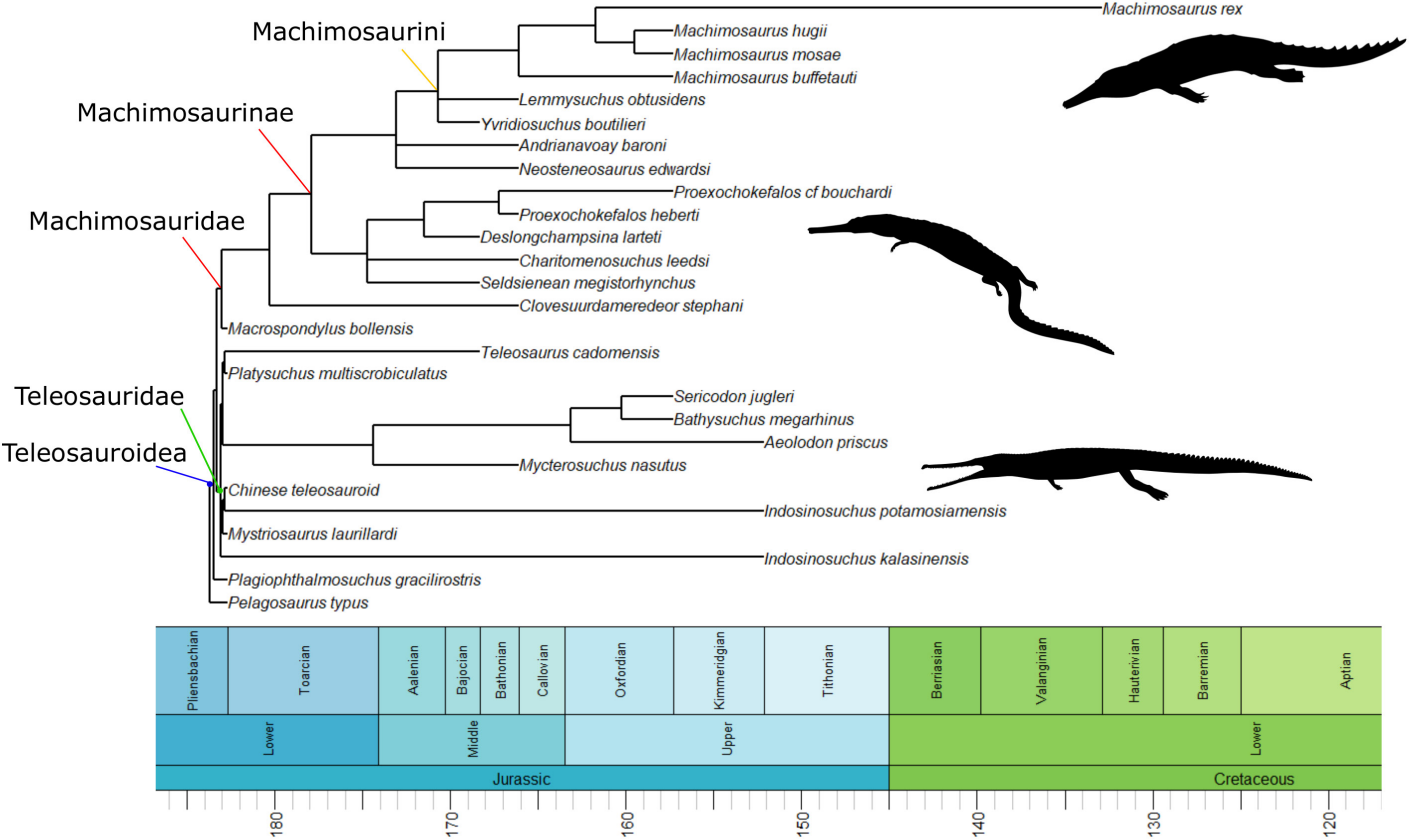
Simplified evolutionary tree and time‐calibrated geological timescale of Teleosauroidea, with the inclusion of *Pelagosaurus typus* (Metriorhynchoidea) as the outgroup. Major clades within Teleosauroidea (Teleosauridae, Machimosauridae, Machimosaurinae and Machimosaurini) are highlighted. Silhouettes provided by PhyloPic© by S. Hartman, G. Monger and N. Tamura.

The anatomy (Andrews, 1913; Eudes‐Deslongchamps, 1867; Foffa et al., 2019; Hua, 1999; Johnson et al., 2017, 2019; Jouve, 2009; Morel de Glasville, 1876; Sachs et al., 2019; Vignaud, 1995; Westphal, 1961, 1962) and more recently the alpha taxonomy and systematics (Figure 1; see Johnson, 2019; Johnson et al., 2020 for more information) of teleosauroids are now well studied, but their hypothesized feeding ecologies and multi‐taxic niche partitioning are still poorly understood. A brief ecological investigation of thalattosuchian palaeobiology was presented by Hua (1997) and Hua and Buffetaut (1997) but this was not discussed in greater detail. Most teleosauroids were considered conservative in morphology (Andrews, 1913; Buffetaut, 1982) and to have occupied similar niches, excluding members from the tribe Machimosaurini due to their robust, massive skeleton and blunt, rounded teeth (Johnson et al., 2017; Young et al., 2014). However, in terms of osteology teleosauroids have recently been shown to display six distinct ecomorphotypes based on skull shape, dentition and postcranial morphology (see Table 1 in Johnson et al., 2020 for more detailed information): longirostrine specialist (e.g., laterally facing orbits); longirostrine generalist; longirostrine semi‐terrestrial form (e.g., large, heavily ornamented dorsal osteoderm “shield”); mesorostrine generalist; durophage/macrophage (e.g., blunt rounded teeth); and longirostrine pelagic form (e.g., reduced forelimbs and osteoderms). In addition, their ecology has never been examined using a quantitative approach.

**TABLE 1:** List of continuous (C) and discrete (D) morphological characters used to characterize teleosauroid dentition (modified from Foffa et al., 2018).

Character type	Description
Continuous (C)	1. General
	C1 = Apicobasal crown height
	C2 = Crown ratio
	C3 = Lingual‐labial curvature
	C4 = Crown angle
Discrete (D)	1. General
	D1 = Labial‐lingual compression
	D12 = Shape of tooth crown apex
	D13 = Non‐procumbent or procumbent dentition
	2. Ornamentation
	D6 = Presence of anastomosed pattern
	D7 = Enamel ornamentation, lingual side
	D8 = Enamel ornamentation, labial side
	D9 = Enamel ridges, relief
	D11 = Texture of enamel
	3. Carinae and/or serrations
	D2 = Presence and size of true denticles
	D3 = Presence or absence of functionally serrated edges
	D4 = Denticle distribution
	D5 = Presence of “pseudodenticles”
	D10 = Presence or absence of false denticles

*Note*: See Data S3 for more detailed descriptions of characters.

Here, we rectify this gap and examine the dentition (the most common marine reptile fossils) and mandibular characteristics to evaluate the feeding ecology of teleosauroids, using quantitative methodology as in Foffa et al. (2018) and Foffa (2018). Notably, we expand the teleosauroid dataset substantially from that used in Foffa et al. (2018) and Foffa (2018) for a more comprehensive, in‐depth evaluation of their feeding ecology.

### 1.1 Institutional abbreviations

GPIT: Paläontologische Sammlung der Eberhard Karls Universität, Tübingen, Germany; MNHN: Muséum National d'Histoire Naturelle, Paris, France; NHMUK: Natural History Museum, London, UK; PETMG: Peterborough Museum and Art Gallery, Peterborough, UK; PRC: Palaeontological Research and Education Centre, Maha Sarakham University, Thailand; SMNS: Staatliches Museum für Naturkunde, Stuttgart, Baden Württemberg, Germany (see Data S1 for additional institutions in dataset).

## 2 MATERIALS AND METHODS

### 2.1 Dataset

We compiled a list of 17 functionally applicable anatomical characteristics of the dentition (Table 1) scored for 38 (approximately 23 species) and 16 functionally applicable mandibular characteristics (Table 2) scored for 18 (approximately 14 species) teleosauroid specimens (Data S1). These datasets were kept separated to both enable comparisons and detect possible lags in evolution between the mandible and dentition (see Foffa, 2018). In addition, multiple teleosauroid tooth specimens were more readily available than complete mandible specimens, which furthered our intention to keep the datasets separate to avoid possible skewed results. The teleosauroid specimens in the datasets are sampled across their entire evolutionary history, from the Early Jurassic (*Plagiophthalmosuchus gracilirostris*: lower Toarcian) to the Early Cretaceous (*Machimosaurus rex*: late Hauterivian/early Barremian) (Data S2). Thus, the specimens come from a wide array of localities and lithological facies, with representatives from four different habitats: freshwater, implied semi‐terrestrial, coastal marine and lagoonal/pelagic (see Data S2 for more details). This extensive range of taxa and environments allows for an overall greater evaluation and understanding of teleosauroid ecology as a group.

**TABLE 2:** List of continuous mandibular measurements

Continuous mandibular character (C)	Description
C1	Mandible length (ML)
C2	Relative length of the symphyseal mandibular area (MSL/ML)
C3	Relative depth of the symphyseal area (MSD/ML)
C4	Depth at the posterior end of the tooth row (eTRD/ML)
C5	Depth at the coronoid process (CPD/ML)
C6	Average mandibular depth (avg MD)
C7	Relative length of the tooth row (TRL/ML)
C8	Relative length of the retroarticular process (RPL/ML)
C9	Anterior mechanical advantage (aMA)
C10	Posterior mechanical advantage (pMA)
C11	Opening mechanical advantage (oMA)
C12	Muscle adductor size (maL/ML)
C13	Gullet size (ASDm/ML)
C14	Relative width of tooth row (eTRW/ML)
C15	Tooth index (TI = 10 × CH/ML)
C16	Tooth index 2 (TI2 = CH/ASDm)

*Note*: See Data S3 for more detailed information.

For the dentition dataset, we scored four continuous and 13 discrete characters for each specimen (Data S3; Table 1), modified from Foffa et al. (2018). Teleosauroids display homodont dentition across the entirety of the mandible; therefore, all tooth crowns in our dataset are the largest tooth found in the anterior section of the tooth row, as in Foffa et al. (2018), for consistency. For the mandible dataset, we scored 16 continuous characters (Data S3; Table 2) for near‐complete or completely preserved mandibles, using the methods found in Foffa (2018). Measurements were taken directly from specimens using digital calipers, excluding curvature and crown angles (C3 and C4; Data S1) and verified on photographs using ImageJ (Schneider et al., 2012). Dental curvature and crown angles were measured using the angle tool in ImageJ (Abramoff et al., 2004; Schneider et al., 2012).

The jaws of crocodylomorphs (and indeed all tetrapods with a simple jaw joint) act as a simple lever for both opening and closing processes (Ballell et al., 2019; Bestwick et al., 2021; Cleuren & Vree, 2000; Sinclair & Alexander, 1987). The efficacy of such lever can be evaluated using mechanical advantage. In simple levers, such as jaw‐systems, mechanical advantage (MA) is the ratio of in‐lever length (moment arm of the muscle) divided by out‐lever length (distance from the jaw condyle to the biting point) and indicates the proportion of muscle adductor force is transmitted at the bite point (Greaves, 1983; Morales‐García et al., 2021; Radinsky, 1981; Stubbs et al., 2013; Westneat, 2003). It is important to note that this metric does not take into account size and that teleosauroids have a large range of values due to the significant variation in snout length and supratemporal muscle size (the influence of size in feeding behavior are further discussed below).

### 2.2 Multivariate analyses

Before analyses, all continuous characters of both tooth and mandible datasets, were standardized using z‐transformation (distributions were equalized to the same mean value, *μ* = 0, and standard deviation, *σ* = 1; Foffa et al., 2018; Stubbs & Benton, 2016) to account for size variation. Both taxon‐character matrices (Data S1) were then transformed into a Gower distance matrix, which allows for the combination of ordered discrete and continuous characters (Gower, 1971). The dental dataset was subjected to both a Principal Component Analysis (PC) and Principal Coordinates Analysis (PCo) in PAST v4.06 (Hammer et al., 2001) following Foffa et al. (2018) and the mandibular dataset was subjected to a PC analysis, to ordinate taxa and produce a plotted morphospace, based on the first two axes (PC1 and PC2 and PCo1 and PCo2, respectively) which represented the highest variation. We included a PCo analysis for the dental dataset as this type of analysis is useful when dealing with discrete characters (Zuur et al., 2007). The mandibular dataset was run a second time with the removal of mandibular length (ML) to assess whether this character influenced the results.

### 2.3 Evolutionary analyses in relation to phylogeny

A simple time‐calibrated phylogenetic tree, centered on a comprehensive, updated phylogenetic analysis of Teleosauroidea (Johnson et al., 2020) was generated in RStudio v3.4.2 using the R packages phytools 0.6 (R Core Team, 2020; Revell, 2012) and ape 4.1 (Paradis et al., 2004) (Data S4). Function DatePhylo (method = “equal”) of the package strap (Bell & Lloyd, 2015) was used to calculate branch lengths. Five ecologically important continuous mandibular features were estimated and mapped on the phylogeny using the fastAnc and contmap (continuous variable map) functions in the R package phytools 0.6 (Revell, 2012): length of mandibular symphysis (MSL/ML), size of muscle attachments (maL/ML) opening mechanical advantage (oMA), anterior mechanical advantage (aMA) and posterior mechanical advantage (pMA) (Data S4). These five characters were chosen for three main reasons: (1) they have distinct biomechanical meaning; (2) are compatible together and characterize functional mandibular properties; and (3) represent simple lever mechanics (Anderson et al., 2011; Anderson & Friedman, 2012; Stubbs et al., 2013). In these analyses, the anterior–posterior length of muscle attachments (maL/ML; which can be measured in extinct taxa) is used as proxy for adductor muscle force (Busbey, 1989; Porro et al., 2011; Sellers et al., 2017). For each feature, the phylogeny was pruned of the tips for which said feature is unavailable.

## 3 RESULTS

### 3.1 Dentition and mandible

**FIGURE 2:**
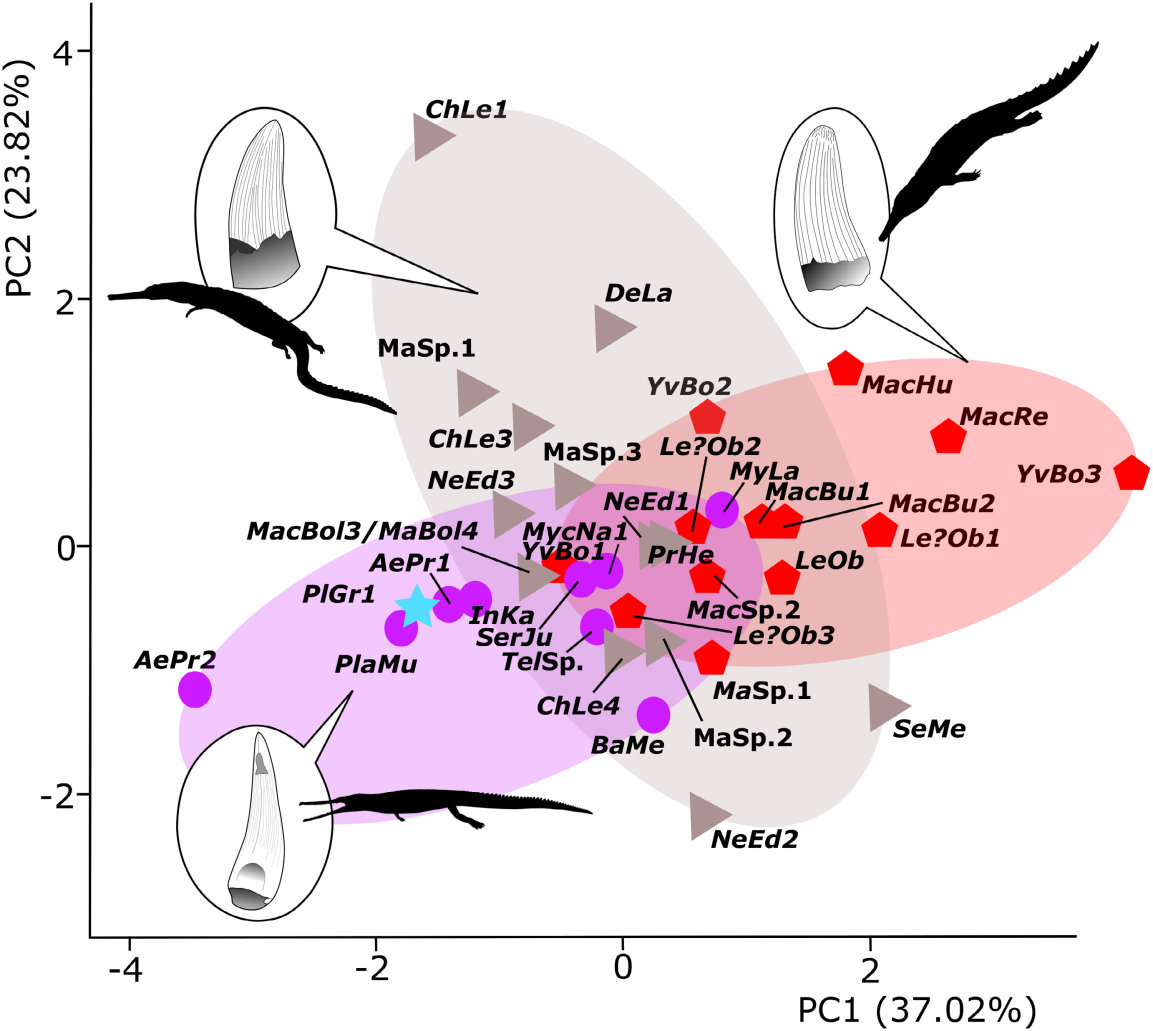
Principal component analysis (PC) of teleosauroid dentition along the PC1 (37.02%) and PC2 (23.82%). The blue star represents the most basal teleosauroid, *Plagiophthalmosuchus gracilirostris*, purple circles represent Teleosauridae, gray triangles indicate Machimosauridae, and red hexagons represent Machimosaurini (a distinctive tribe within Machimosauridae). See Data S2 for abbreviated names. Silhouettes provided by PhyloPic© by S. Hartman, G. Monger and N. Tamura.

PC1 is largely related to the presence of pseudodenticles, anastomosed pattern and apex shape (37.02%) while PC2 is largely associated with apicobasal crown length (23.82%) (Figure 2). PC1 and PC2 (Figure 2) show that machimosaurin specimens (*Yvridiosuchus*, *Lemmysuchus*, *Machimosaurus*) are clustered together and largely distinct from all other teleosauroid taxa; this is due to their distinctive tooth characteristics, such as their conical shape, blunt apices, and pronounced enamel ornamentation (composed of numerous tightly packed ridges in the basal and mid‐crown regions, but an anastomosed pattern at the apex) (Young et al., 2014, 2015). In contrast, there is greater overlap between teleosaurids and non‐machimosaurin machimosaurids (Figure 2). In general, the dentitions of these groups are relatively similar (despite distinct separation in phylogenetic terms): the tooth crowns are long and slender with a slight lingual curvature, their apices are sharp, and the enamel ridges are faint. There are three exceptions: the teleosaurid *Mystriosaurus* (NHMUK PV OR 14781) and the machimosaurids *Neosteneosaurus* (PETMG R178) and *Proexochokefalos* (MNHN.F 1890‐13). In these genera, the largest teeth are robust and well ornamented but retain a relatively sharp apex with no apical enamel ornamentation. The basal‐most teleosauroid, *Plagiophthalmosuchus*, is nestled amongst teleosaurids and is closely positioned to *Platysuchus* (SMNS 9330) (Figure 2). Overall, these results are consistent with those found in Foffa et al. (2018), in which machimosaurins were also clearly separated from other teleosauroids. In PC2 (23.82%) and PC3 (21.24%), there is massive overlap between all teleosauroid taxa. Aside from the dentition in Machimosaurini, the results do not correspond to the six osteological ecomorphotypes (Johnson et al., 2020) discussed above.

**FIGURE 3:**
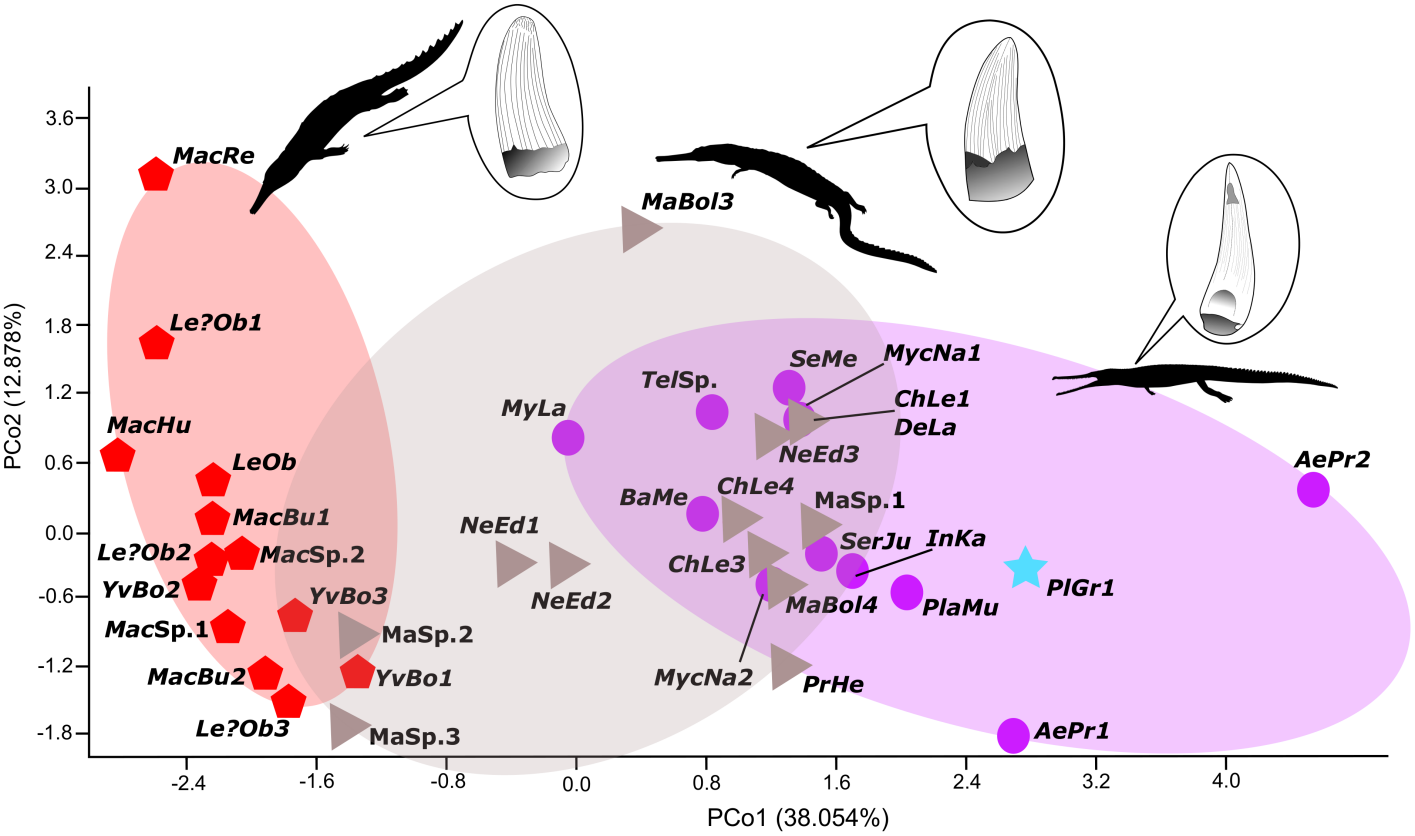
Principal coordinates analysis (PCo) of teleosauroid dentition along the PCo1 (38.05%) and PCo2 (12.88%). The blue star represents the most basal teleosauroid, *Plagiophthalmosuchus gracilirostris*, purple circles represent Teleosauridae, gray triangles indicate Machimosauridae, and red hexagons represent Machimosaurini (a distinctive tribe within Machimosauridae). See Data S2 for abbreviated names. Silhouettes provided by PhyloPic© by S. Hartman, G. Monger and N. Tamura.

PCo1 is largely related to dental ornamentation, apex shape, and tooth curvature (38.05%) and PCo2 is described as apicobasal crown height (12.88%) (Figure 3). As with the PC analysis, machimosaurins (*Yvridiosuchus*, *Lemmysuchus*, *Machimosaurus*) are closely clustered together, whereas other teleosauroids show greater overlap with one another (Figure 3).

**FIGURE 4:**
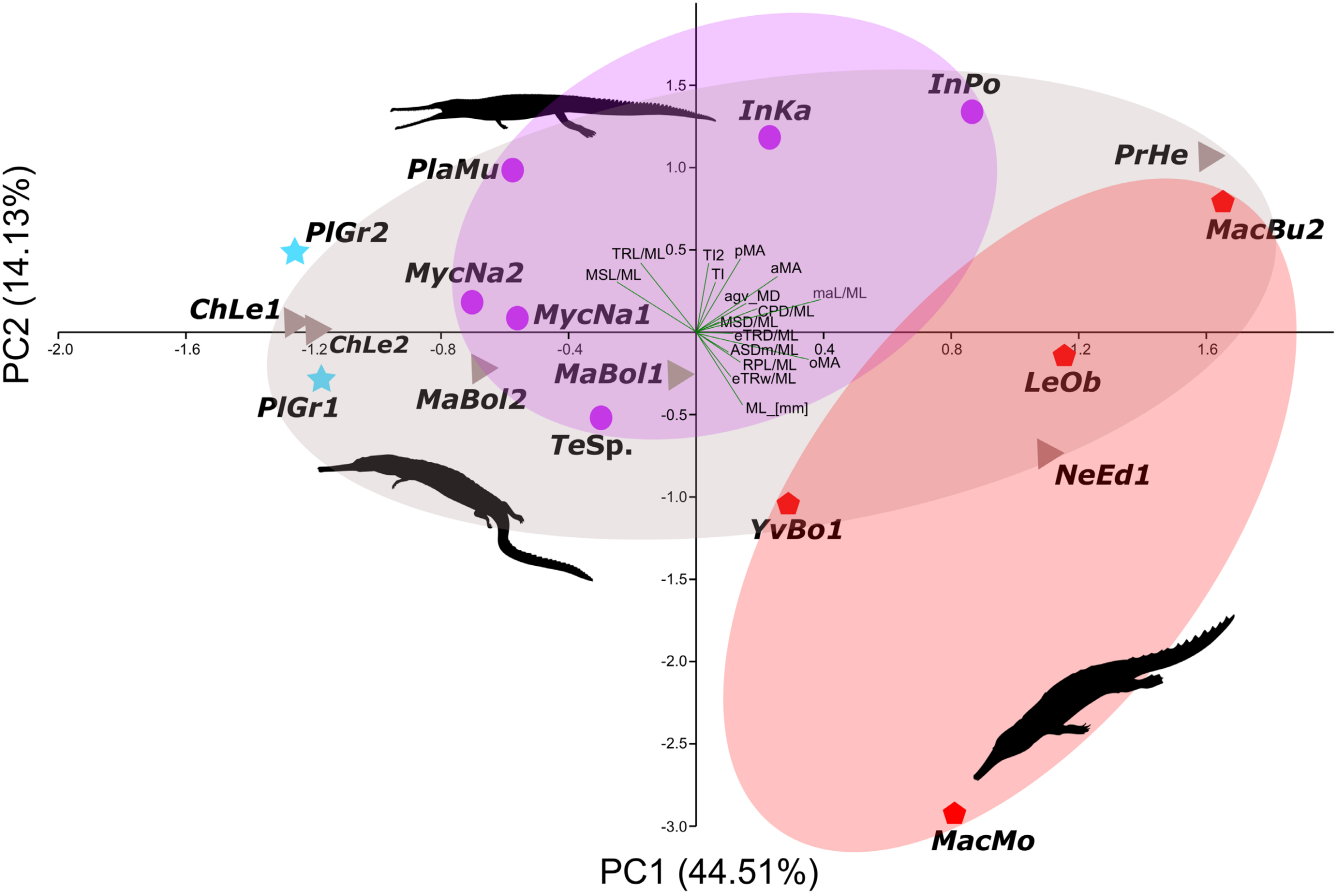
Principal component analysis (PCA) of teleosauroid mandibles along the PCA1 (44.51%) and PCA2 (14.13%). The blue stars represent specimens of the most basal teleosauroid, *Plagiophthalmosuchus gracilirostris*, purple circles represent Teleosauridae, gray triangles indicate Machimosauridae, and red hexagons represent Machimosaurini (a distinctive tribe within Machimosauridae). See Data S2 for abbreviated names. Silhouettes provided by PhyloPic© by S. Hartman, G. Monger and N. Tamura.

In our mandibular analysis (Figure 4), PC1 is largely associated with mandibular length (ML) and muscle attachment size (maL) (44.51%) while PC2 is largely associated with mandibular symphysis length (MSL) and tooth index (14.13%). *Plagiophthalmosuchus* and most teleosaurids (e.g., *Mycterosuchus*) cluster negatively along PC1 (Figure 3), which is also the case in basal machimosaurids (e.g., *Macrospondylus*, *Charitomenosuchus*). However, *Indosinosuchus potamosiamensis* (PRC‐11) and *I. kalasinensis* (PRC‐239) are separated from other teleosaurids; both are positioned positively along PC1 and PC2, possibly due to a slightly shorter mandible. The majority of non‐machimosaurin machimosaurids range negatively along PC1 and PC2 (Figure 4); only *Proexochokefalos* and *Neosteneosaurus* place positively along PC1. Machimosaurins (*Yvridiosuchus*, *Lemmysuchus*, and *Machimosaurus*) cluster together along negative PC1 and positive PC2, aside from *Mac. buffetauti* (which is both positive along PC1 and PC2). *Neosteneosaurus* (which is placed phylogenetically closest to machimosaurins; see Johnson et al., 2020), is nearest to machimosaurins along both PC1 and PC2 (Figure 4). When mandibular length was removed, the overall distribution of the taxa in the morphospace did not change. As with the dentition, the results of the mandibular analysis do not correspond to the six osteological ecomorphotypes (Johnson et al., 2020) discussed above.

### 3.2 Evolutionary analysis

**FIGURE 5:**
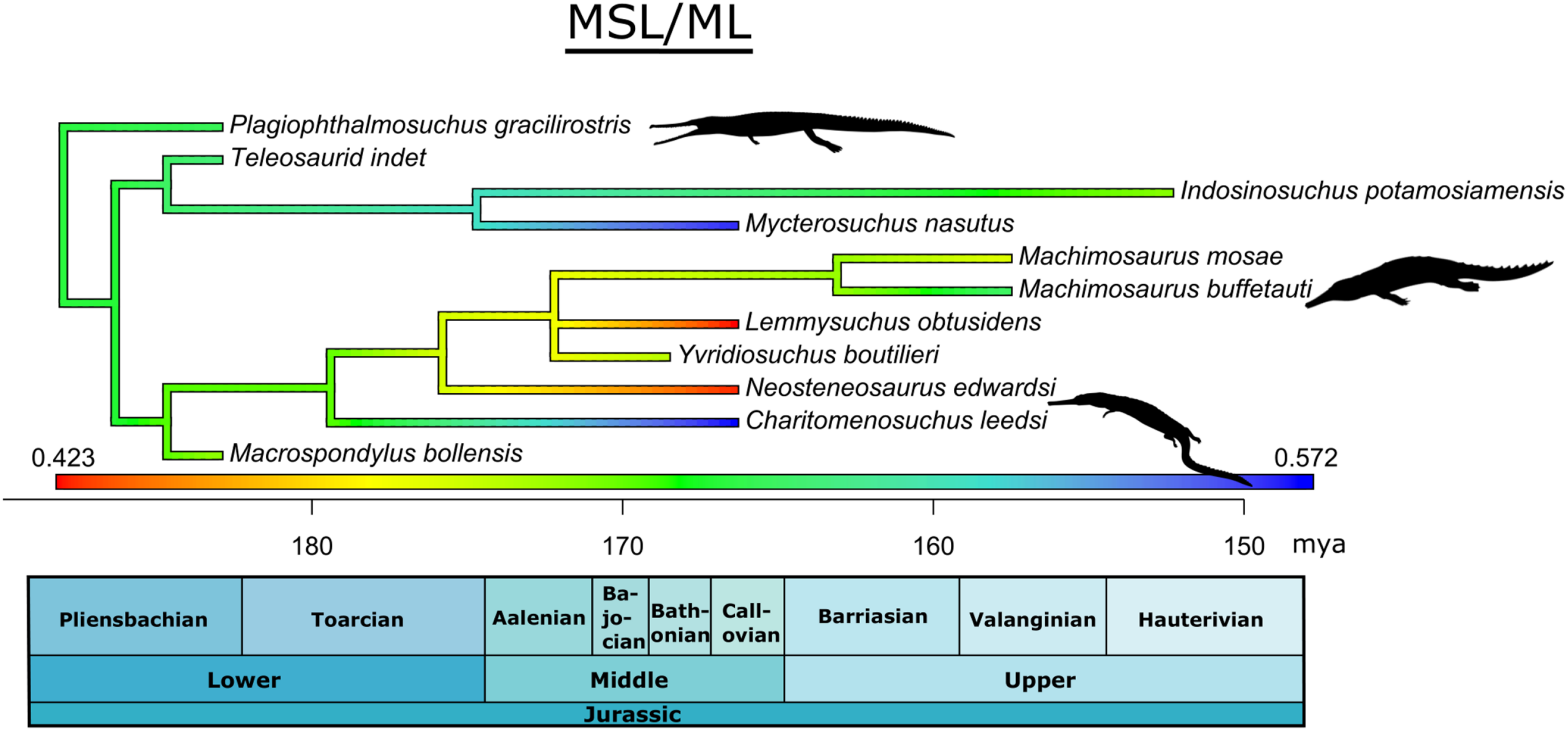
Simplified teleosauroid evolutionary tree with time‐calibrated scale and heatmap displaying length of the mandibular symphysis relative to the mandibular ramus length (MSL/ML). Silhouettes provided by PhyloPic© by S. Hartman, G. Monger and N. Tamura.

The length of the mandibular symphysis relative to the mandibular ramus length (MSL/ML) is linked with the tolerance of biomechanical loads and bite forces (Holliday & Nesbitt, 2013; Iordansky, 1963, 1973; Lessner et al., 2019). Within teleosauroids, the teleosaurid *Mycterosuchus* and the machimosaurid *Charitomenosuchus* display the longest mandibular ramus length relative to mandibular length (0.56 and 0.65, respectively), whereas the machimosaurine machimosaurids have the shortest (e.g., 0.42 for *Lemmysuchus*) (Figure 5). *Plagiophthalmosuchus* (0.50), *I. potamosiamensis* (0.48), *Mac. buffetauti* (0.50) and *Mystriosaurus* sp. (NHMUK PV R 5703; 0.51) all have a relatively intermediate mandibular ramus length values (Figure 5).

**FIGURE 6:**
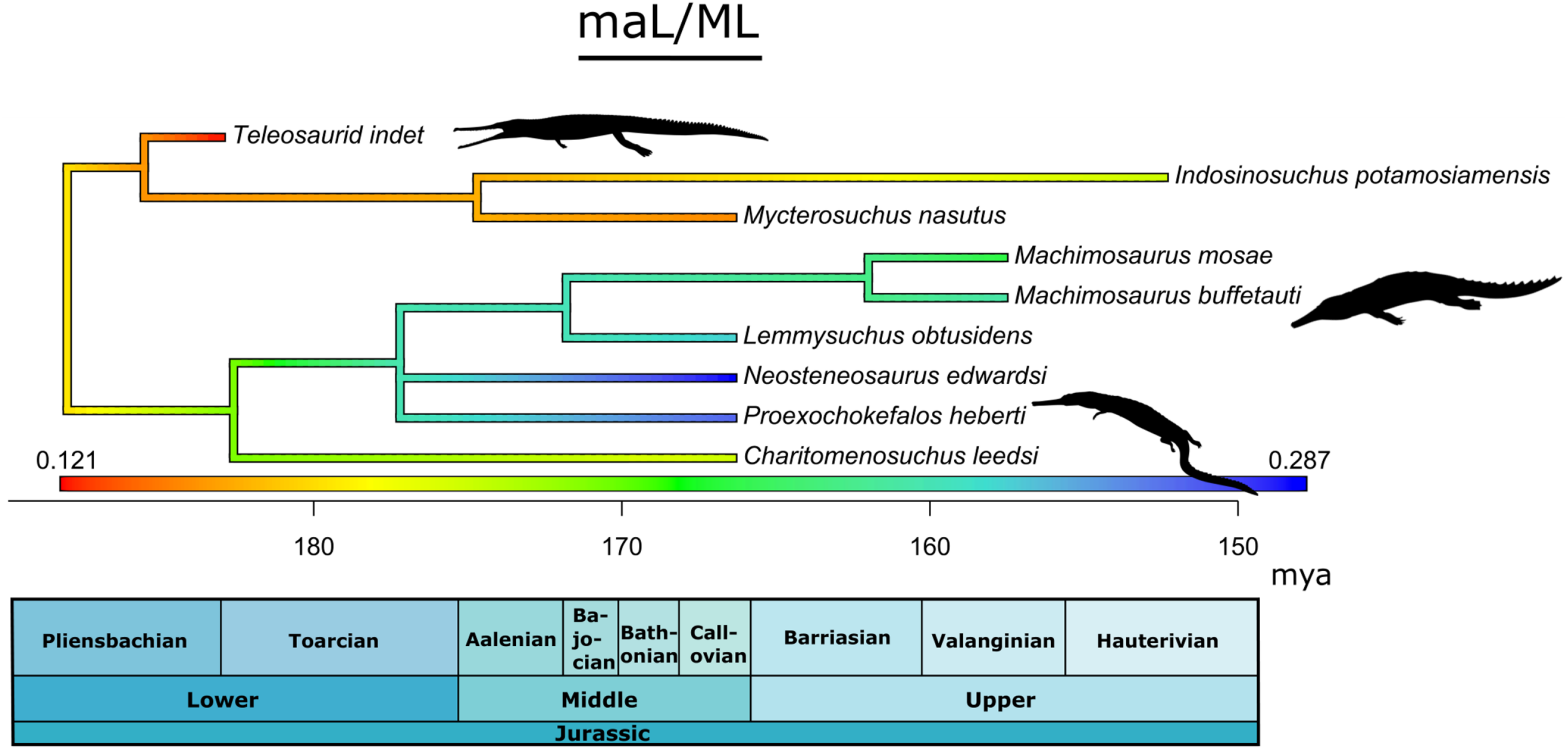
Simplified teleosauroid evolutionary tree with time‐calibrated scale and heatmap displaying length of the adductor muscle attachment sites relative to the mandibular ramus length (maL/ML). Silhouettes provided by PhyloPic© by S. Hartman, G. Monger and N. Tamura.

The length of the adductor muscle attachment sites relative to mandibular ramus length (maL/ML) is related to bite force (Busbey, 1989; Porro et al., 2011; Sellers et al., 2017). The machimosaurids *Proexochokefalos* and *Neosteneosaurus* have the largest (relative to jaw length) muscle attachment sites (0.27 and 0.28, respectively), even more so than most machimosaurins (see Section 4) (Foffa, 2018). *Lemmysuchus* has relatively large muscle attachment sites (0.25), slightly larger than *Mac. mosae* (0.21) and *Yvridiosuchus* (0.21) (Figure 5). *Mystriosaurus* sp. (NHMUK PV R 5703) has the shortest muscle attachment sites (0.13), followed by the teleosaurid *Mycterosuchus* (0.14) and the machimosaurid *Charitomenosuchus* (NHMUK PV R 3806; 0.14) (Figure 6). The teleosaurid *I. potamosiamensis* has a slightly lower value than *Lemmysuchus* (0.24).

**FIGURE 7:**
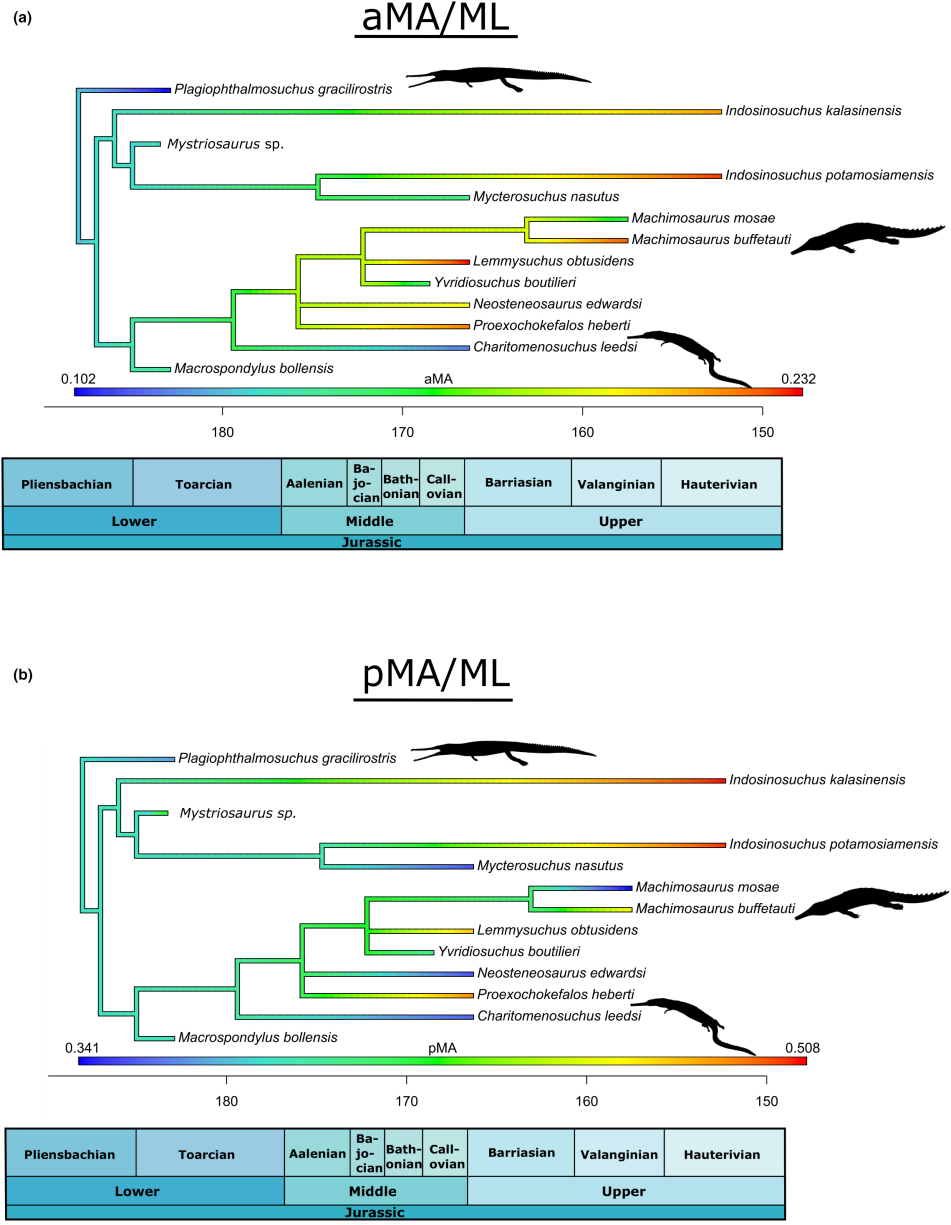
Simplified teleosauroid evolutionary tree with time‐calibrated scale and heatmap displaying (a) anterior mechanical advantage (aMA) and (b) posterior mechanical advantage (pMA). Silhouettes provided by PhyloPic© by S. Hartman, G. Monger and N. Tamura.

Anterior mechanical advantage (aMA) (Figure 7a) evaluates the minimum value of mechanical advantage along the tooth row, or the amount of input (muscle) force transmitted to the anterior bite positions (Foffa, 2018). This metric was important to measure because teleosauroids have enlarged fang‐like teeth, which were presumably involved in prey capturing, at the anterior end of their rostrum. Both *Indosinosuchus* taxa have the two of the highest anterior mechanical advantage (*I. potamosiamensis*: 0.22; *I. kalasinensis*: 0.21), along with *Lemmysuchus* (0.23), *Proexochokefalos* (0.22) and *Mac. buffetauti* (0.22). *Plagiophthalmosuchus* and *Mystriosaurus* sp. have the lowest anterior mechanical advantage values (0.10 and 0.11, respectively). In contrast, posterior mechanical advantage (pMA) (Figure 7b) evaluates the maximum value of mechanical advantage along the tooth row (Foffa, 2018). The teleosaurid *I. potamosiamensis* has the highest posterior mechanical advantage (0.50) closely followed by *Proexochokefalos* (0.48), *Mac. buffetauti* (0.46) and *Lemmysuchus* (0.47). *Charitomenosuchus* (NHMUK PV R 3320; 0.32), *Mac. mosae* (0.34), and *Mystriosaurus* sp. (NHMUK PV R 5703; 0.34) have the lowest posterior mechanical advantage values, whereas *Plagiophthalmosuchus* (0.37), *Yvridiosuchus* (0.40) and *Mycterosuchus* (0.39) have more intermediate values.

**FIGURE 8:**
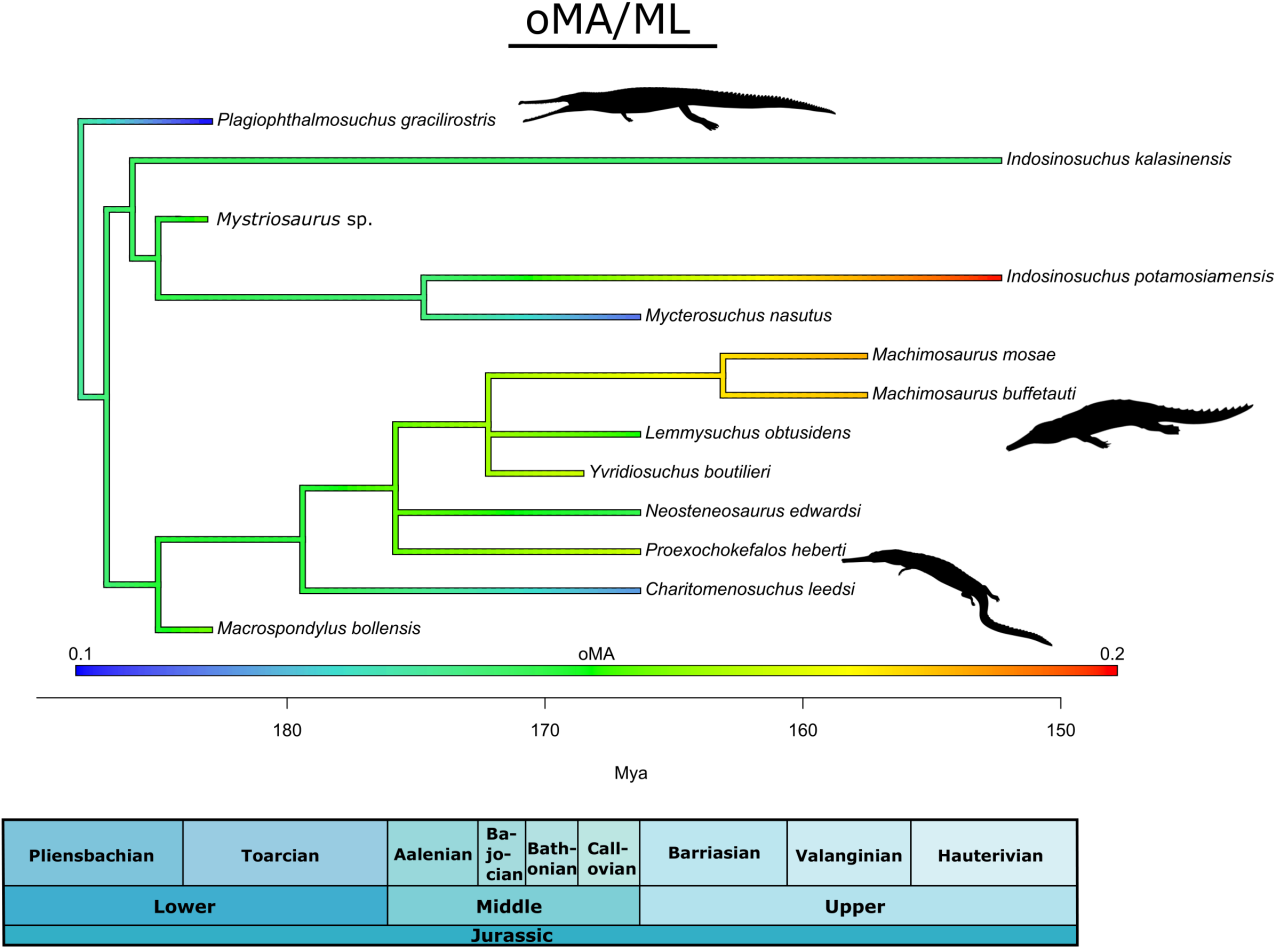
Simplified teleosauroid evolutionary tree with time‐calibrated scale and heatmap displaying opening mechanical advantage (oMA). Silhouettes provided by PhyloPic© by S. Hartman, G. Monger and N. Tamura.

Opening mechanical advantage (oMA) is a measure of maximum jaw opening/closing speed (Foffa, 2018; Stubbs et al., 2013). Lower values indicate of a relatively “faster” bite. *Plagiophthalmosuchus*, the most basal teleosauroid, has the lowest oMA of all teleosauroids (NHMUK PV OR 15500; 0.10) (Figure 8), followed by the teleosaurid *Mycterosuchus* (0.11) and machimosaurid *Charitomenosuchus* (NHMUK PV R 3320; 0.11) (Figure 8). The teleosaurid *I. potamosiamensis* has the highest opening mechanical advantage (0.2); *Mac. mosae* (0.18) and *Mac. buffetauti* (0.18). *Lemmysuchus* (0.15), *Yvridiosuchus* (0.16), *Proexochokefalos* (0.16) and *Mystriosaurus* sp. (NHMUK PV R 5703; 0.14) all have relatively intermediate oMA values, whereas the teleosaurid *I. kalasinensis* (0.13) has a slightly lower opening mechanical advantage value (Figure 8).

The results show that, in general, teleosauroid mandibles (aside from Machimosaurini) perform similarly regardless of phylogenetic position. There is little variation in long‐snouted forms (most teleosaurids and basal machimosaurids); however, derived non‐machimosaurine machimosaurids exhibit a gradual shift to mandibles with larger muscles attachment sites, a shorter mandibular symphysis and more robust, deep jaw. Machimosaurins then show both a mandible and dental set well adapted for macrophagy/durophagy (see Section 4 below).

## 4 DISCUSSION

### 4.1 Biomechanical implications

With regards to our tooth analyses Machimosaurini mostly separate from all other teleosauroids along both PC1 and PC2 and PCo1 and PCo2, consistent with the results in Foffa et al. (2018). This result is expected, given the distinctive tooth morphology of machimosaurins compared with other teleosauroids (e.g., pronounced enamel ornamentation including an apical anastomosed pattern, conical shape, and blunt apex) (Johnson et al., 2017; Young et al., 2014, 2015). Non‐machimosaurin machimosaurids were spread out across PCA2, whereas most teleosaurids were restricted to the negative PC1 and PC2 regions of morphospace; however, there was significant overlap between these two groups, regardless of habitat, location or geological age. Our results suggest that groups other than machimosaurins may have had overlapping feeding strategies, despite different habitats and osteological skull and mandibular features. The teleosaurid *Mystriosaurus* and the machimosaurid *Neosteneosaurus* are situated most closely to Machimosaurini along PC1 (Figure 2), which may be due to these taxa having large, robust teeth while maintaining a relatively pointed apex.

**FIGURE 9:**
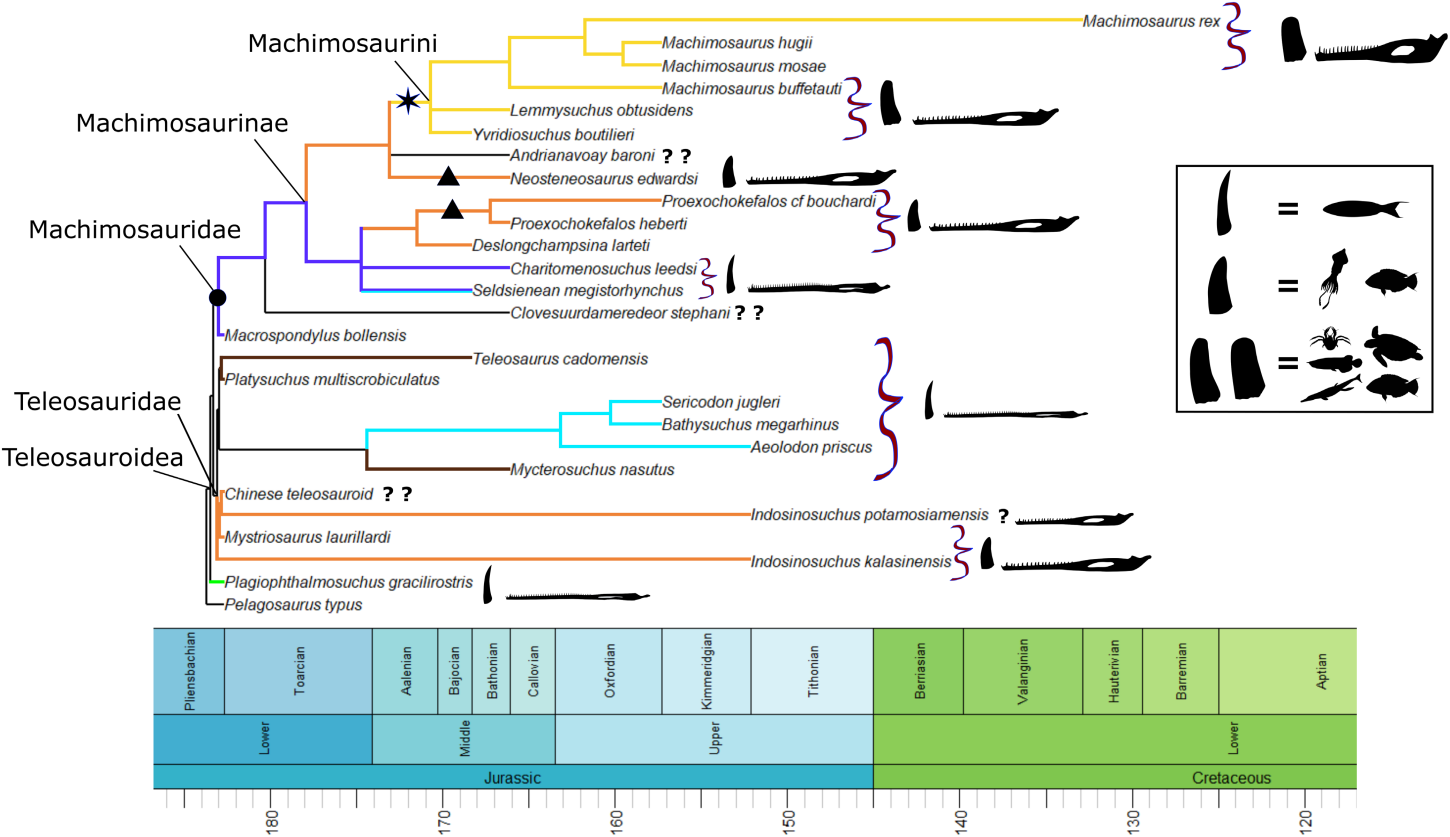
Simplified teleosauroid evolutionary tree with time‐calibrated geological scale displaying six different ecomorphotypes within Teleosauroidea and different ecotype divergences within Machimosauridae. For ecomorphotypes: green represents longirostrine specialist; light blue represents pelagic form; yellow represents macrophage/durophage form; brown represents semi‐terrestrial form; purple represents longirostrine generalist; orange represents mesorostrine generalist; and black represents unknown. For machimosaurid ecotypes: circle represents ecotype 1; triangle represents ecotype 2; star represents ecotype 3 (with [left] corresponding tooth and [right] mandible silhouettes, in which a question mark represents unknown). The box shows hypothesized prey items. Silhouettes provided by PhyloPic© by Spotila, K. Sorgan, I. Braasch, E. Schumacher, C. Cevrim, and H. Filhol.

**FIGURE 10:**
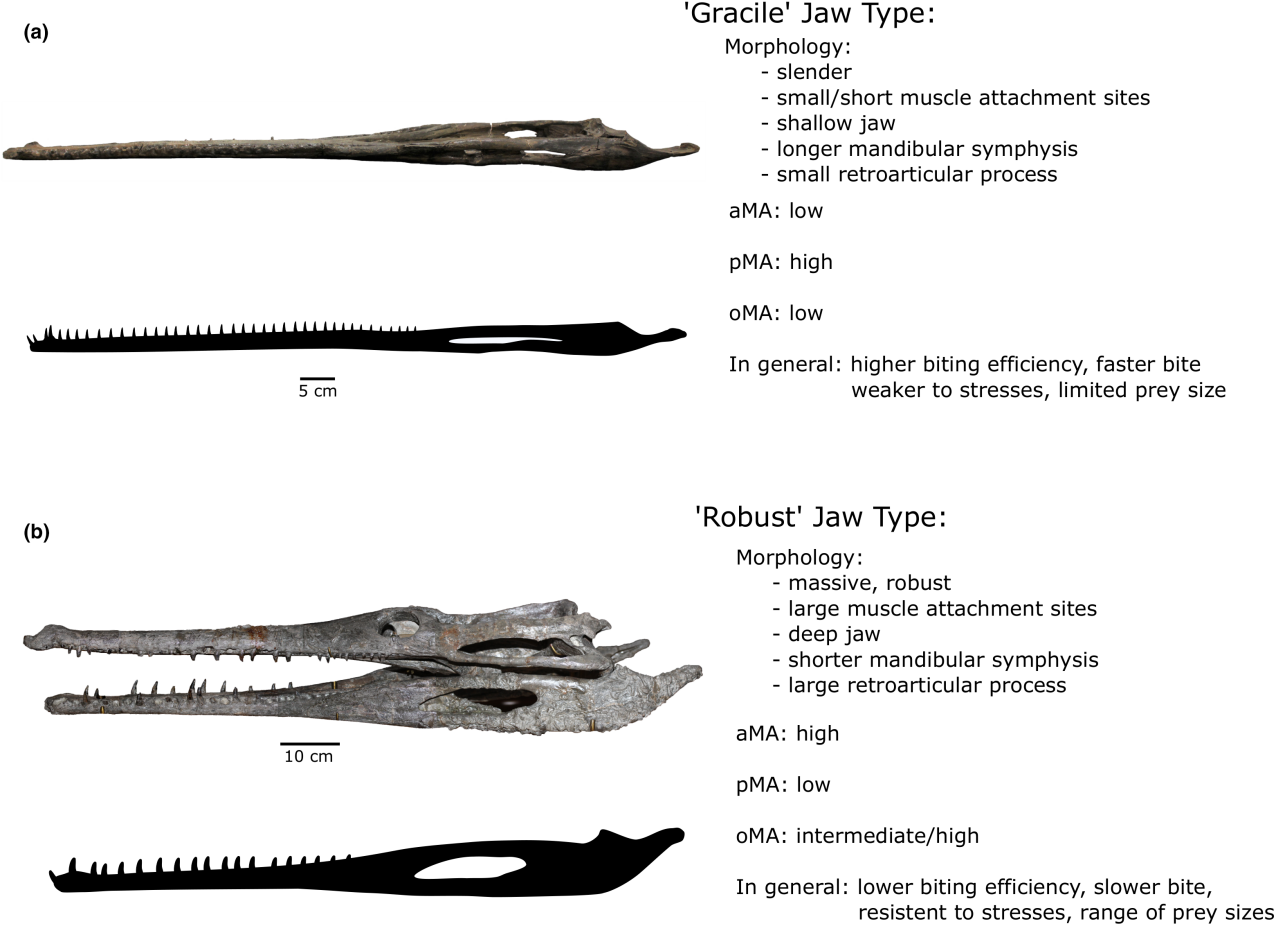
Visualization of the two different jaw types within teleosauroids: (a) the “gracile jaw type” and (b) the “‘robust jaw type”. Specimen and silhouette for the gracile type is *Charitomenosuchus leedsi* (NHMUK PV R 3806), and specimen and silhouette for the robust type is *Proexochokefalos heberti* (MNHN.F 1890‐13). Scale bars: 5 cm (a) and 10 cm (b).

In our mandibular results, there is a clear evolutionary trend along PC1 from slender mandibles with relatively small adductor muscles (low maL/ML) and short muscle attachment sites (“gracile jaw type”; Figures 9 and 10a) to shorter, broader mandibles with relatively large muscle attachment sites (high maL/ML) (“robust jaw type”; Figures 9 and 10b). Mechanically, small muscle attachment site values generally allow for a higher biting efficiency due to the last tooth being closer to the mandibular musculature; the long distance of the out‐lever arm of the opening mechanical advantage (oMA) ultimately produces a faster bite. The “gracile jaw type” therefore provides a larger surface area for puncturing prey when biting, increasing the speed of attack and prey capture success rate (Ballell et al., 2019; Pierce et al., 2008; Stubbs et al., 2021; Taylor, 1987). A relatively long tooth row often, but not always, corresponds to a shorter adductor muscle attachment size which contributes to an overall weaker bite (Stubbs et al., 2021).

“Gracile” jaws (Figure Figures 9 and 10a) can also experience, and have reduced resistance to, increased stress, torsion and bending during feeding (Ballell et al., 2019; Walmsley et al., 2013), which can limit prey options. It is important to note, however, that relative head size (relative to prey size) may greatly influence how resistant an individual is to stresses. For example, the Indian gharial (*Gavialis gangeticus*) has a long, tubular snout with the weakest bite out of all living crocodylians (Erickson et al., 2012); however, individuals with skull lengths of approximately 1 m are capable of preying upon birds and large mammals (Ballell et al., 2019; Thorbjarnarson, 1990), and their lower jaws are structurally resistant, capable of feeding on large loads (Ballell et al., 2019). Overall size is a key factor that influences how strong an individual's bite is and the types of prey they can consume, as discussed below. Within teleosauroids, the “gracile jaw type” (slender; high efficiency; fast but weak bite) is present in *Plagiophthalmosuchus*, most teleosaurids and early diverging non‐machimosaurine machimosaurids (*Macrospondylus* and *Charitomenosuchus*) (Figure 2). These taxa also have the least optimized out‐lever in the lower jaw. The anterior jaw is where maximal loads are dealt with (Wroe et al., 2005) and is therefore important when processing prey items. *Plagiophthalmosuchus*, most teleosaurids, and smaller individuals of *Macrospondylus* and *Charitomenosuchus* display a relatively weaker anterior mechanical advantage (aMA), suggesting that, while they were able to quickly grab prey items, it may have taken time to properly subdue and process them.

In contrast, the “robust jaw type” (e.g., shorter mandibular symphysis and deeper mandibular rami) (Figures 9 and 10b) are mechanically more resistant to certain stresses when consuming harder prey items (Pierce et al., 2009; Stubbs et al., 2021). “Robust” jaws generally have a higher anterior bite efficiency, lower posterior bite efficiency and limited biting surface area, as the last tooth is further away from the fulcrum of the jaw (articular surface). However, massive, brevirostrine/mesorostrine jaws can compensate for this by increasing the MA of the adductor muscles; this produces an overall stronger bite where the symphyseal region is resistant to multiple types of stresses, which is advantageous for subduing prey (Morales‐García et al., 2021; Pierce et al., 2009). Higher bite forces can contribute to less time handling and processing prey items (Verwaijen et al., 2002), and in modern crocodylians, a shorter mandibular symphysis performs well when dealing with heavier loads (McCurry et al., 2015; Walmsley et al., 2013). In *Machimosaurus*, the mandibular symphysis has been considerably shortened in comparison with other teleosauroids and adductor muscle attachment sites (maL) are exceptionally large. Machimosaurin mandibles, particularly in *Machimosaurus*, are characterized by: (1) enlarged adductor musculature; (2) short mandibular symphyses; and (3) robustness (Young, Brusatte, Beatty, et al., 2012; Young, Brusatte, de Andrade, et al., 2012). This combination of features allows for an efficient anterior bite, as more of the muscle forces are converted into bite forces, but at the cost of reducing jaw opening speed (Taylor, 1987).

The curvature of the posterior portion of the mandible also provides insight into biomechanical adaptations. In machimosaurins (*Yvridiosuchus*, *Lemmysuchus* and *Machimosaurus*), the posterior half of the lower jaw is sharply dorsally curved (Johnson et al., 2017). This may be due to three possible adaptations for increasing bite force: (1) enlarging the size of muscle attachment sites; (2) re‐orientating the *pterygoideus* muscles; and (3) increasing gape. In addition, retroarticular process length and orientation are crucial to bite force, as it is the insertion site for two important jaw muscles (*musculus depressor mandibulae* and *musculus pterygoideus ventralis*; Holliday et al., 2013) and acts as a major anatomical in‐lever in crocodylomorphs (Gignac & O'Brien, 2016). Machimosaurins have shortened, laterally broad, dorsally curved retroarticular processes, which increases space for the *m. depressor mandibulae* and *m. pterygoideus ventralis*. This combination of a dorsally curved mandible and broad retroarticular process increases the insertion site for and modifying the line of action of the *musculus pterygoideus*, in conjunction with increasing optimum gape angles (Figure 10).

Biting performance decreases as gape increases (Herring & Herring, 1974; Jessop et al., 2006), and therefore macropredatory taxa tend to exhibit adaptations for higher biting performances at wider gapes (Herring & Herring, 1974). A wider gape is also needed when consuming larger prey items. This is observed in metriorhynchids such as *Dakosaurus*, *Tyrannoneustes* and *Plesiosuchus* (Foffa & Young, 2014; Young, Brusatte, Beatty, et al., 2012; Young, Brusatte, de Andrade, et al., 2012), which exhibit three main characteristics that infer increased performance during wide gape biting; (1) shortening the rostrum, which increases MA of the adductors; (2) enlarging the supratemporal fenestrae, which increases adductor muscle force magnitude; and (3) high tooth crown development, which increases shearing surface area (Foffa & Young, 2014; Young et al., 2010, 2013; Young, Brusatte, Beatty, et al., 2012; Young, Brusatte, de Andrade, et al., 2012). Crucially, one key feature that enabled certain teleosauroids, including machimosaurins, to achieve macropredator status was their large body and head sizes, as discussed below.

When referring to opening mechanical advantage (oMA), a low value is indicative of a jaw optimized for closing speed and a high value indicates a jaw specialized for biting force (Morales‐García et al., 2021). Overall, mechanical advantage effectively offers a continuum between velocity and force. It is important to note that extant crocodylians possess hypertrophied *pterygoideus*, allowing for fast closure of the jaws and very high bite forces. However, the muscular architecture of thalattosuchians was probably quite different compared with modern crocodylians; thalattosuchian lateral pterygoid flanges are much smaller, and the *pterygoideus* muscles were likely less developed than in Crocodylia. In general, *Plagiophthalmosuchus* and teleosaurids have a lower opening mechanical advantage and anterior mechanical advantage and higher posterior mechanical advantage, indicating jaws optimized for closing quickly (Figures 7 and 8). In general, derived machimosaurids (particularly the machimosaurins) have a higher opening mechanical advantage and anterior mechanical advantage and lower posterior mechanical advantage, signifying jaws that close slowly but with heavy force behind them.

### 4.2 Teleosauroid evolutionary ecology

Overall, our analyses show that the mandibles of both Teleosauridae and Machimosauridae (excluding Machimosaurini) performed similarly, suggesting that there was not a major feeding ecology divide between the two groups (Figure 9). This is particularly evident in the long‐snouted forms and presents an interesting parallel with the study of Johnson et al. (2020), in where the authors found multiple distinguishing features within the crania and postcrania of most genera, but relatively few distinctive mandibular characteristics (aside from the dentition). This suggests that, at least in terms of feeding, teleosauroids (excluding Machimosaurini and close relatives) remained relatively conservative, with limited mandibular functional diversity.

Overall, the mandibles of most teleosaurids and basal machimosaurids do not show any significant differences, as most taxa retained an elongated, slender mandible with pointed teeth that was ideal for catching small, fast prey (Figure 9; Drumheller & Wilberg, 2020). It is curious that while no great variation is observed in mandibular mechanics amongst the long‐snouted forms, many of them (particularly teleosaurid taxa) were living in different habitats, such as semi‐marine (e.g., *Charitomenosuchus*), pelagic (e.g., *Aeolodon*), freshwater (e.g., *Indosinosuchus*) and more terrestrial (e.g., *Platysuchus*) (Foffa et al., 2019; Johnson et al., 2020; Martin et al., 2019). This suggests the possibility that teleosaurids and basal machimosaurids where generally either feeding in a similar manner or on similar prey types but in different habitats, and that habitat preference, in addition to snout length and size, was likely a major driver in resource partitioning, rather than mandibular functionality. Amongst these long‐snouted taxa, *Mycterosuchus* exhibits an optimal jaw type for catching fast‐moving prey. A combination of an extremely elongated mandible, small muscle attachments and comparatively low opening mechanical advantage, as well as slender, curved, pointed teeth, suggest that it was specialized in catching quick prey items such as fishes. However, and intriguingly, within teleosaurids *Indosinosuchus* taxa are more closely positioned to basal machimosaurids on PC1 (see Figure 5). This may be due to these taxa having a slightly shorter and deeper jaw than other teleosaurids. In addition, the two *Indosinosuchus* species in the dataset have divergent opening mechanical advantage (oMA) values (Figure 8): *I. kalasinensis* has much lower oMA value (0.13) than *I. potamosiamensis* (0.2), despite anterior mechanical advantage, posterior mechanical advantage and muscle attachment site values being relatively similar for both taxa. This is particularly interesting, as *Indosinosuchus* taxa are only known from the same freshwater deposits in the Late Jurassic lower Phu Kradung Formation in northeastern Thailand (Johnson et al., 2020; Martin et al., 2019), and a differing oMA could possibly suggest nice partitioning within teleosauroid species found in the same environment.

As mentioned previously, long‐snouted basal machimosaurids (e.g., *Macrospondylus*, *Charitomenosuchus*) exhibit similar feeding styles to teleosaurids, but derived non‐machimosaurine machimosaurids (e.g., *Proexochokefalos* and *Neosteneosaurus*) show signs of the mandible switching to a diet not necessarily requiring speed or high bite efficiency but rather capable of subduing larger, specialized prey (e.g., increased musculature, shortening and posterodorsal curvature of the jaw, stress resistant). These taxa also compensated for their relatively slower bite, low bite efficiency and limited biting space by having shortened and robust jaws and increased muscle adductor areas, which were better suited for feeding on potentially slower but more heavily armored prey. Our analyses suggest that there were three machimosaurid ecotypes (Figure 9): (1) basal machimosaurids (e.g., *Macrospondylus*) that were biomechanically similar to teleosaurids; (2) derived machimosaurids *Proexochokefalos* and *Neosteneosaurus*, in which the mandible was adapted for hard‐bodied prey, but the dentition still retained certain *Macrospondylus*‐like features (e.g., no apical anastomosed ornamentation, curvature at the tooth apex); and (3) Machimosaurini (*Yvridiosuchus*, *Lemmysuchus* and *Machimosaurus*), where both the mandible and dentition were adapted for feeding on armored prey. Interestingly, in any given ecosystem only one representative of each of these three machimosaurid groups was numerically dominant, with the others being either rare or absent. For example, in the Middle Jurassic Oxford Clay Formation (OCF), *Neosteneosaurus* (ecotype 2) and *Charitomenosuchus* (ecotype 1) are common but *Lemmysuchus* (ecotype 3) and *Proexochokefalos* (ecotype 2) taxa are relatively scarce. In the Late Jurassic, *Machimosaurus* (ecotype 3) is dominant in terms of both absolute abundance and species richness, while *Proexochokefalos* cf. *bouchardi* (ecotype 2) is extremely rare (Johnson et al., 2020). In addition, machimosaurin taxa made up for a relatively slower and lower biting efficiency by growing to large sizes, as discussed below.

As discussed previously, *Proexochokefalos* had a mandible well adapted for tackling large prey, with some of the largest muscle attachment sites (shared with *Neosteneosaurus*) and opening mechanical advantage within teleosauroids (Figure 7), near equal to *Machimosaurus*. Importantly, Vignaud (1995), Foffa (2018) and Foffa et al. (2018) noted that *Proexochokefalos* displays an intermediate tooth morphology between the standard “longirostrine” species and *Machimosaurus* (e.g., moderately labiolingually flattened, pointed apices and modest enamel ornamentation). It may possibly be linked to an intermediate phase in which this taxon experimented with catching a diverse array of prey that were more difficult to catch (further experimentation features might include enlarged basioccipital tuberosities and head dorsiflexion musculature characteristic to this taxon). However, a different hypothesis could be that this represents another distinct machimosaurid feeding ecology. *Proexochokefalos* and *Neosteneosaurus* both had large skulls, with relatively robust teeth, and enlarged adductor musculature. It is possible that there are two trends of macrophagy within Machimosaurinae: one leading to *Machimosaurus* and is a macrophagy/durophagy suite, and another more generalized macrophagy that combined large size with intermediate, less specialized dentition (exhibited by *Proexochokefalos* and *Neosteneosaurus*).

During the Late Jurassic, there was a diverse assemblage of eucryptodiran turtles (Anquetin et al., 2014; Joyce et al., 2021; Püntener et al., 2015), particularly in Europe. Bite marks and embedded teeth suggest that *Lemmysuchus* and *Machimosaurus* specialized in macrophagy/durophagy, feeding on larger, armored prey such as turtles and scaled fishes (Meyer, 1988; Young et al., 2014; Young, Brusatte, Beatty, et al., 2012; Young, Brusatte, de Andrade, et al., 2012). It is possible that early machimosaurines began to successfully exploit these prey types, evolving the necessary mandibular tools (short and broad jaws, large muscles, high bite force, and wider gape) to successfully overpower them. Interestingly, our analyses suggest that characteristics toward macrophagy/durophagy in the teleosauroid mandible evolved first (e.g., deep, robust jaws; shortened mandibular symphysis; shortened and curved retroarticular process), with specific tooth characteristics (e.g., blunt apex; little to no curvature; conspicuous enamel ornamentation) evolving afterwards. In certain areas, such as Morocco and Switzerland, machimosaurids are found alongside turtle plastrons with machimosaurid teeth embedded in them (Meyer, 1991; Young et al., 2014).

### 4.3 Macrophagy in teleosauroids

Large size is beneficial for macropredation, as it allows an animal to feed upon a multitude of different‐sized prey items (particularly larger and more energetically feasible ones) and reduces the time taken to process prey (Verwaijen et al., 2002). In general, larger animals, as well as animals with large heads, bite harder (Verwaijen et al., 2002) and are more resistant to stresses (Ballell et al., 2019). Large head and body size also compensates for a slower bite or lower biting efficiency by increasing the proportions, strength and mass of an animal. Machimosaurins represent some of the largest teleosauroids in terms of body size, with some *Machimosaurus* taxa reaching over 7 m in length (Young et al., 2016). This implies that, despite a quantitatively slower bite, in absolute terms machimosaurins were still able to seize prey relatively quickly and efficiently due to their massive bulk, in addition to biting harder and processing food quicker.

During teleosauroid evolution, there was an independent shift toward big body size/head size in both teleosaurids and machimosaurids. The teleosaurids *Mycterosuchus* (NHMUK PV R 2617, mandibular length: 1091 mm) and *Mystriosaurus* (NHMUK PV OR 14781, mandibular length at least 911 mm), as well as the early machimosaurid *Macrospondylus* (GPIT‐PV‐31382, mandibular length: approximately 1279 mm), may have been able to bite harder and exploit other prey items than other slender‐snouted taxa because they grew to such large sizes. *Mystriosaurus* in particular displays features superficially similar to machimosaurines, such as a more robust and dorsoventrally deep jaw and intermediate dentition. Known only from the Toarcian, *Mystriosaurus* and *Macrospondylus* show that teleosauroids were already experimenting with pseudo‐macrophagy and large size early on in their evolution.

## 5 CONCLUSION

Historically, the ecology of teleosauroids has been considered conservative (Andrews, 1913; Buffetaut, 1982). However, recent papers discussing specific teleosauroid habitats and osteological ecomorphotypes (Foffa et al., 2018, 2019; Johnson et al., 2020; Martin et al., 2016) show that teleosauroid ecology is more complex than originally thought. We provide an ecological quantitative assessment of teleosauroids by using tooth and mandibular measurements, following the methods used by Foffa (2018) and Foffa et al. (2018). The results of our tooth analysis are similar, but greatly expand to those found in Foffa et al. (2018), in which members of Machimosaurini were clearly separate and all other teleosauroids overlapped with one another. Similarly, our mandibular analyses reveal a much clearer evolutionary trend from: (1) *Plagiophthalmosuchus*, most teleosaurids and basal machimosaurids with a generally long mandibular symphysis, small muscle attachments, faster bite and high bite efficiency to (2) teleosauroids within Machimosaurinae with a generally short mandibular symphysis, large muscle attachments, relatively slower bite and lower bite efficiency. However, machimosaurins and their closely related taxa (*Proexochokefalos* and *Neosteneosaurus*) make up for a lower bite efficiency with increased body size and robusticity. One possible explanation for this extreme change in jaw type is the shift toward larger prey items in Machimosaurinae, ultimately leading to the exploitation of heavily armored prey by Machimosaurini, such as turtles and larger fishes. In addition, an independent preferential shift toward larger head and body size can be seen in both teleosaurids (e.g., *Mycterosuchus*, *Mystriosaurus*) and machimosaurids (e.g., *Macrospondylus*, *Neosteneosaurus*, machimosaurins). Ultimately, there is not a great deal of mandibular variability in teleosaurids and machimosaurids (despite differing habitat preferences in certain taxa), suggesting a subtle feeding ecological divide between the two groups. Resource partitioning was primarily related to snout and skull length as well as habitat; only twice (from ecotype 1 to 2 and ecotype 2 to 3) did teleosauroids manage to make a major evolutionary leap to feed distinctly differently, with only the derived machimosaurines successful in radiating into new feeding ecologies.

## AUTHOR CONTRIBUTIONS

**Michela M. Johnson:** Conceptualization (lead); data curation (equal); formal analysis (lead); funding acquisition (equal); investigation (lead); methodology (equal); visualization (lead); writing – original draft (lead). **Davide Foffa:** Conceptualization (supporting); data curation (equal); formal analysis (supporting); funding acquisition (equal); investigation (supporting); methodology (equal); visualization (supporting); writing – review and editing (supporting). **Mark T. Young:** Supervision (equal); writing – review and editing (supporting). **Stephen L. Brusatte:** Supervision (equal); writing – review and editing (supporting).

## CONFLICT OF INTEREST

The authors declare no competing interests.

### OPEN RESEARCH BADGES

This article has earned an Open Data badge for making publicly available the digitally‐shareable data necessary to reproduce the reported results. The data is available at http://osf.io.

### DATA AVAILABLILITY STATEMENT

The authors declare that all the data supporting the findings of this study are available within the paper and its supplementary data files.

## Supporting information


Data S1

Data S2

Data S3

Data S4
