# Supplementary material for: The ecological diversification and evolution of Teleosauroidea (Crocodylomorpha, Thalattosuchia), with insights into their mandibular biomechanics
Source: Ecol Evol. 2022 Nov 18;12(11):e9484. doi: 10.1002/ece3.9484 (PMC9674474; doi:10.1002/ece3.9484)
Supplement: Supplementary file 2 — Data S2 [file ECE3-12-e9484-s004.docx]

Supplementary Data 2. Comprehensive information of the teleosauroid taxa used in both the tooth and mandibular analyses, including specimen number, taxon abbreviation, inferred habitat and age, as well as which analysis the taxon was used in (T = tooth analysis; M = mandibular analysis; B = both analyses). Hyp = hypothesis/inferred age. Taxa were assigned to four different hypothesized habitats based on literature (in the case of the East Asian freshwater taxa) and certain morphological features: large, tightly interlocked and heavily ornamented dorsal osteoderms indicated semi-terrestrial; and reduced forelimbs and osteoderms indicated pelagic.

| **Taxon** | **Specimen Number** | **Abbreviation** | **Habitat** | **Age** | **Analysis** |
| --- | --- | --- | --- | --- | --- |
| *Plagiophthalmosuchus gracilirostris* | MNHNL TU515  NHMUK PV OR 15500 | *PlGr1*  *PlGr2* | Semi-aquatic | Toarcian | B  M |
| *Mystriosaurus laurillardi* | NHMUK PV OR 14781 | *MyLa* | Semi-aquatic | Toarcian | T |
| *Indosinosuchus potamosiamensis* | PRC-11 | *InPo* | Freshwater | Hyp.: Tithonian | M |
| *Indosinosuchus kalasinensis* | PRC-239 | *InKa* | Freshwater | Hyp.: Tithonian | B |
| *Platysuchus multiscrobiculatus* | SMNS 9930 | *PlaMu* | Semi-terrestrial | Toarcian | B |
| *Teleosaurus* sp. | NHMUK PV R 6377 | *Tel*Sp. | Semi-terrestrial | Bathonian | T |
| *Mycterosuchus nasutus* | NHMUK PV R 2617  CAMSM J.1420 | *MycNa1*  *MycNa2* | Semi-terrestrial | Callovian | B  M |
| *Aeolodon priscus* | MNHN.F.CNJ 78  NHMUK PV R 1086 | *AePr1*  *AePr2* | Pelagic | Tithonian | T  T |
| *Sericodon jugleri* | NRM-PZ R.2337 | *SerJu* | Pelagic | Tithonian | T |
| *Bathysuchus megarhinus* | DORCM G05067 i-v | *BaMe* | Pelagic | Kimmeridgian | T |
| *Macrospondylus bollensis* | MMG BwJ 689  SMNS 51563  SMNS 51957  GPIT-PV-31382 | *MaBol1*  *MaBol2*  *MaBol3*  *MaBol4* | Semi-aquatic | Toarcian | M  M  T  T |
| *Seldsienean megistorhynchus* | MMT P28-1 | *SeMe* | Semi-aquatic | Bathonian | T |
| *Charitomenosuchus leedsi* | NHMUK PV R 3806  NHMUK PV R 3320  BRLSI-GP1770a-e  LPP.M.37 | *ChLe1*  *ChLe2*  *ChLe3*  *ChLe4* | Semi-aquatic | Callovian | B  B  T  T |
| *Deslongchampsina larteti* | OUMNH J.29851 | *DeLa* | Semi-aquatic | Bathonian | T |
| *Proexochokefalos heberti* | MNHN.F 1890-13 | *PrHe* | Semi-aquatic | Callovian | B |
| *Neosteneosaurus edwardsi* | PETMG R178  NHMUK PV R 2865  NHMUK PV R3701 | *NeEd1*  *NeEd2*  *NeEd3* | Semi-aquatic | Callovian | B  T  T |
| *Yvridiosuchus boutilieri* | OUMNH J.29850  NHMUK PV OR 28611  NHMUK PV OR 40127 | *YvBo1*  *YvBo2*  *YvBo3* | Semi-aquatic | Bathonian | B  T  T |
| *Lemmysuchus obtusidens* | NUMUK PV R3168 | *LeOb* | Semi-aquatic | Callovian | B |
| *Lemmysuchus* cf. *obtusidens* | CAMSM J65408  DORCM G3939  OUMNH J40669 | *Le*?*Ob1*  *Le?Ob2*  *Le?Ob3* | Semi-aquatic | Callovian | T  T  T |
| *Machimosaurus buffetauti* | DFMMh F330  SMNS 91415 | *MacBu1*  *MacBu2* | Semi-aquatic | Kimmeridgian | T  B |
| *Machimosaurus mosae* | Plastotype | *MacMo* | Semi-aquatic/open ocean | Tithonian | B |
| *Machimosaurus hugii* | MG-25 | *MacHu* | Semi-aquatic | Kimmeridgian | T |
| *Machimosaurus rex* | ONM NG 5 | *MacRe* | Semi-aquatic | Hauterivian | T |
| *Machimosaurus* sp. | MJML K839  GPIT-RE-03037 | *Mac*Sp.1  *Mac*Sp.2 | Semi-aquatic | Kimmeridgian/  Tithonian | T  T |
| Teleosaurid sp. | NHMUK PV R 5703 | TeSp. | Semi-aquatic | Toarcian | M |
| Machimosaurid sp. | MNHN specimen (no number)  NHMUK PV R 4764  NHMUK PV OR 47161 | MaSp.1  MaSp.2  MaSp.3 | Semi-aquatic | Callovian  Bathonian | T  T  T |
