# Supplementary material for: The ecological diversification and evolution of Teleosauroidea (Crocodylomorpha, Thalattosuchia), with insights into their mandibular biomechanics
Source: Ecol Evol. 2022 Nov 18;12(11):e9484. doi: 10.1002/ece3.9484 (PMC9674474; doi:10.1002/ece3.9484)
Supplement: Supplementary file 3 — Data S3 [file ECE3-12-e9484-s001.docx]

Supplementary Data 3. Detailed description of continuous (C) and discrete (D) characters for both the (1) dentition and (2) mandibular analyses.

*1. Continuous and discrete characters used in the dental analyses. These were modeled after Foffa (2018) and Foffa et al. (2018).*

**C1: Apicobasal crown height (CH).** This character is defined as the length (in a straight line/tangent plane) from the apex to the crown-root juncture.

**C2: Crown ratio (CR).** This character is defined as the ratio between the apicobasal lengths of the crown (C1) divided by the mean mesial-distal diameter of the crown base.

**C3: Lingual-labial curvature (LLcufrv).** This character is the ratio between the length of the labial surface divided by the length of the medial face of the tooth crown. Measurements are taken in the mesiodistal direction along the crown margins. Strongly curved crowns have high values (> 1), while the value of 1 indicates perfectly conical teeth. The measurements were taken in the canonical anterior/posterior view of high resolution photographs of the specimens using ImageJ.

**C4: Crown angle (CAngle).** This character is defined as the angle measured on the largest dentary tooth in labial/lingual view. This was done using high-resolution photographs in ImageJ.

**D1:** Labial-lingual compression. Labial-lingual compression of the tooth: absent (0), weakly compressed (1) or strongly compressed (2). Modified from Young et al., 2016, Ch.184.

**D2:** Presence and size of true denticles: absent (0), incipient microziphodonty (1), conspicuous microziphodonty (2) or macroziphodonty (3). Note, the terms ‘microziphodonty’ and ‘macroziphodonty’ follows that of Foffa et al. (2017). Modified from Young et al. 2016, Ch. 215.

**D3:** Presence or absence of functionally serrated edges: absent (0) or present (1).

**D4:** Denticle distribution: non-contiguous (0) or contiguous (1). Modified from Young et al. 2016, Ch.216.

**D5:** Presence of ‘pseudodenticles’: absent (0) or present (1).

**D6:** Presence of anastomosed pattern: absent (0) or present (1). Anastomosed pattern refer to detailed, branching crenulations of enamel ridges on the apex of the tooth. Modified from Young et al. 2016, Ch.221.

**D7:** Enamel ornamentation, lingual side: absent (0), largely absent, or present with weak apicobasal ridges (1), present and consists of numerous, spaced defined apicobasal ridges (2) or present and consists of conspicuous, numerous, well-defined, closely packed apicobasal ridges (3). Modified from Foffa et al., 2018.

**D8:** Enamel ornamentation, labial side: largely absent, or present with weak apicobasal ridges (0), present and consists of numerous, spaced defined apicobasal ridges (1) or present and consists of conspicuous, numerous, well-defined, closely packed apicobasal ridges (2). Modified from Foffa et al., 2018.

**D9:** Enamel ridges, relief: absent or extremely low (absent macroscopically) (0), low relief but macroscopically distinct, <0.5 mm (1) or medium relief, <1 mm (2). Modified from Foffa et al., 2018.

**D10:** Presence or absence of false denticles: absent (0) or present (1). Denticles refer to ornamentation that interferes with the carinae. Modified from Young et al. 2016, Ch.214.

**D11:** Texture of enamel: smooth (0) or pebbled (1).

**D12:** Shape of tooth crown apex: sharp and pointed (0) or blunt and round (1). Modified from Young et al. 2016, Ch.206.

**D13:** Non-procumbent or procumbent dentition: non-procumbent (0) or procumbent (1).

*2. Continuous characters used in the mandibular analyses. These were modeled after Foffa (2018).*

**C1: Total mandible length (ML).** This character was measured from the anterior-most dentary (premaxilla) to the posterior-most part of the dentary (retroarticular process), a direct line from one another. Note that *Mystriosaurus laurillardi* (NHMUK PV OR 14781) was excluded from this analysis because most of the retroarticular process was missing and therefore the total mandible length could not be accurately calculated.

**C2: Relative length of the symphyseal mandibular area (MSL/ML).** Relative length of the mandibular symphysis. This character was measured in dorsal/ventral view from the anterior-most dentary to the posterior-most area of the mandibular symphysis. During biting, the symphyseal area experiences major stresses and its length determines the mechanical response of the entire ramus, with a long symphysis being mechanically less resistant to shaking and twisting. Teleosauroid taxa with more elongate mandibles (e.g., *Charitomenosuchus leedsi*: NHMUK PV R 3806) generally have higher MSL/ML values than taxa with shorter mandibles (e.g., *Machimosaurus mosae*: plastotype)

**C3: Relative depth of the symphyseal area (MSD/ML).** Relative depth of the symphyseal area. In combination with the previous character, mandibular symphysis depth determines the mechanical resistance of the most stressed area of the lower jaw during feeding. This character, along with C4, C5 and C6, serves as a proxy for the cross-section of key sections of each mandibular ramus. The cross-section (and material dispersal within it) determines the resistance to bending and torsion of a structure; a deeper section has improved resistance to bending than a thinner one. In teleosauroids, the mandibular symphyseal relative depth is highest in *Machimosaurus buffetauti* SMNS 91415 (~4.9%) and lowest in *Plagiophthalmosuchus gracilirostris* MNHNL TU515 (~1.0%).

**C4: Depth at the posterior end of the tooth row (eTRD/ML).** Similar to C3, this character evaluates the resistance of the mandibular ramus, focusing particularly on the posterior area. In *Proexochokefalos heberti* (MNHN.F 1890-13) and *Neosteneosaurus edwardsi* (PETMG R178), the posterior area deepens drastically (~6.6%).

**C5: Depth at the coronoid process (CPD/ML).** This character measures the deepest point of the lower jaws. In teleosauroids, this character was measured at the deepest part of the posterior mandible.

**C6: Average mandibular depth (avg MD).** This character estimates average depth of the mandibular ramus across its entire length. In teleosauroids, the character was measured (in lateral view) by taking the mandibular volume and dividing it by the length of the mandible squared (ML^2^).

**C7: Relative length of the tooth row (TRL/ML).** The relative length of the total tooth row, from first alveolus to last alveolus. This is an important character since it (1) controls the stretching or shortening of the out-lever arms, which in turn influences variation in bite speed and forces (see Anderson et al. 2011; Stubbs et al. 2013), and (2) increases the available rostral length for capturing prey. The variation of this character has been associated to piscivory and macrophagy (Young et al., 2012b). In teleosauroids, *Plagiophthalmosuchus gracilirostris* NHMK PV OR 15500 has the longest tooth row (~73%) and the *Machimosaurus mosae* plastotype has the shortest (~44%).

**C8: Relative length of the retroarticular process (RPL/ML).** The posterior-most area of the mandible is an important attachment site for several key muscles associated with opening and closing of the jaws. In general, machimosaurids (particularly machimosaurins) have a longer retroarticular processes, while teleosaurids have shorter ones.

**C9: Anterior mechanical advantage (aMA).** Mechanical advantage value at the first dentary alveolus. This character represents the minimum value of MA along the tooth row. The in-lever is represented by the distance from the fulcrum (quadrate-articular surface) to the anterior-most dentary tooth/alveolus, while the out-lever is the distance from the articular surface and the middle of the adductor muscle attachment site (anterior retroarticular process). In teleosauroids, *Lemmysuchus obtusdiens* NHMUK PV R 3168 and *Indosinosuchus potamosiamensis* PRC-11 have the highest aMA value (~0.23) and *Charitomenosuchus leedsi* NHMUK PV R 3806 has the lowest value (~0.11).

**C10: Posterior mechanical advantage (pMA).** Mechanical advantage value at the posterior most dentary alveolus. This character represents the maximum value of MA along the tooth row. The in-lever is represented by the distance from the fulcrum (quadrate-articular surface) to the posterior-most dentary tooth, while the out-lever is the distance from the articular surface and the middle of the adductor muscle attachment site (anterior retroarticular process). In teleosauroids, *Indosinosuchus* taxa (PRC-11 and PRC-239) have the highest pMA vale (~0.50), while *Charitomenosuchus leedsi* NHMUK PV R 3320 has the lowest value (~0.32).

**C11: Opening mechanical advantage (oMA).** This character was measured at the anterior-most tooth position to evaluate the minimum value (e.g., maximum jaw closing speed). The in-lever is represented by the distance between the fulcrum (quadrate-articular surface) and the posterior end of the lower jaw, while the out-lever is the distance between the fulcrum (quadrate-articular surface) and the anterior-most dentary tooth. In teleosauroids, *Plagiophthalmosuchus* (MNHNL TU515 and NHMUK PV OR 15500) has the lowest oMA (~0.10) and *Machimosaurus* taxa (SMNS 91415 and plastotype) have the highest (~0.18).

**C12: Muscle adductor size (maL/ML).** The relative length of the adductor muscle attachment sites in relation to mandibular ramus length. This character examines the size of the adductor muscle attachment sites and was used as a proxy for adductor muscle force. In teleosauroids, *Machimosaurus buffetauti* SMNS 91415 and *Neosteneosaurus edwardsi* PETMG R178 have the longest maL/ML (~0.28) and the indeterminate teleosaurid NHMUK PV R 5703 has the shortest (~0.13).

**C13: Gullet size (ASDm/ML).** This character was measured as the distance between the articular surfaces (divided by mandibular length), and represents the maximum prey size that an animal is able to swallow. In teleosauroids, *Machimosaurus mosae* plastotype has the largest gullet size (~0.21) and *Charitomenosuchus leedsi* NHMUK PV R 3806 has the smallest (~0.11).

**C14: Relative width of tooth row (eTRW/ML).** This character was measured as the relative width between the posterior-most dentary alveoli. It was used in the dataset as it has been found significant in extant marine tetrapods. In general, teleosaurids have a smaller eTRW/ML value (~0.10 to 0.13) than machimosaurids (greater than 0.40).

**C15: Tooth index 1 (TI = 10*CH/ML).** This first tooth index is evaluated as the ratio between 10x the largest crown apicobasal length (CH) divided ML. The largest tooth used for this character was the fourth dentary tooth. In teleosauroids, *Proexochokefalos heberti* MNHN.F 1890-13 has the greatest 10*CH/ML value (~0.39) and *Plagiophthalmosuchus gracilirostris* MNHNL TU515 had the smallest value (~0.12).

**C16: Tooth index 2 (TI = CH/ASDm).** Largest crown apicobasal length (CH) divided relative to gullet size. As with the previous character, this compares the size of the largest tooth with the gullet size. In teleosauroids, *Machimosaurus buffetauti* SMNS 91415 and *Proexochokefalos heberti* MNHN.F 1890-13 have the largest CH/ASDm value (~0.19) and the *Machimosaurus mosae* plastotype has the lowest value (~0.08).
